# Supplementary material for: Kawasaki Disease Patient Stratification and Pathway Analysis Based on Host Transcriptomic and Proteomic Profiles
Source: Int J Mol Sci. 2021 May 26;22(11):5655. doi: 10.3390/ijms22115655 (PMC8198135; doi:10.3390/ijms22115655)
Supplement: Supplementary file 1 [file ijms-22-05655-s001.zip › Supplementary Figures.pdf]

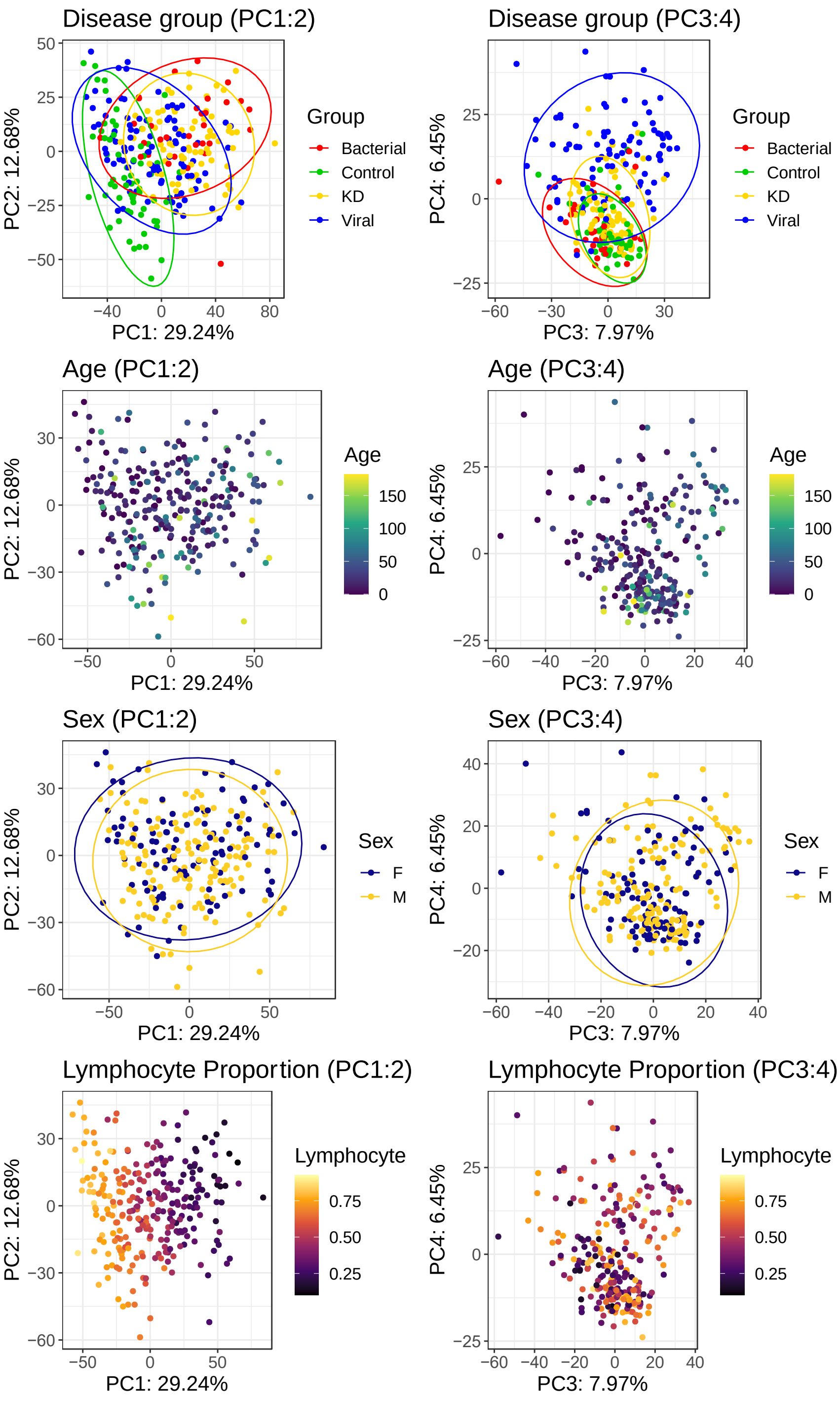

Figure S1. Principal component analysis (PCA) plots for the transcriptomic dataset with points coloured by disease group, age, sex and lymphocyte proportion for principal components 1:4.

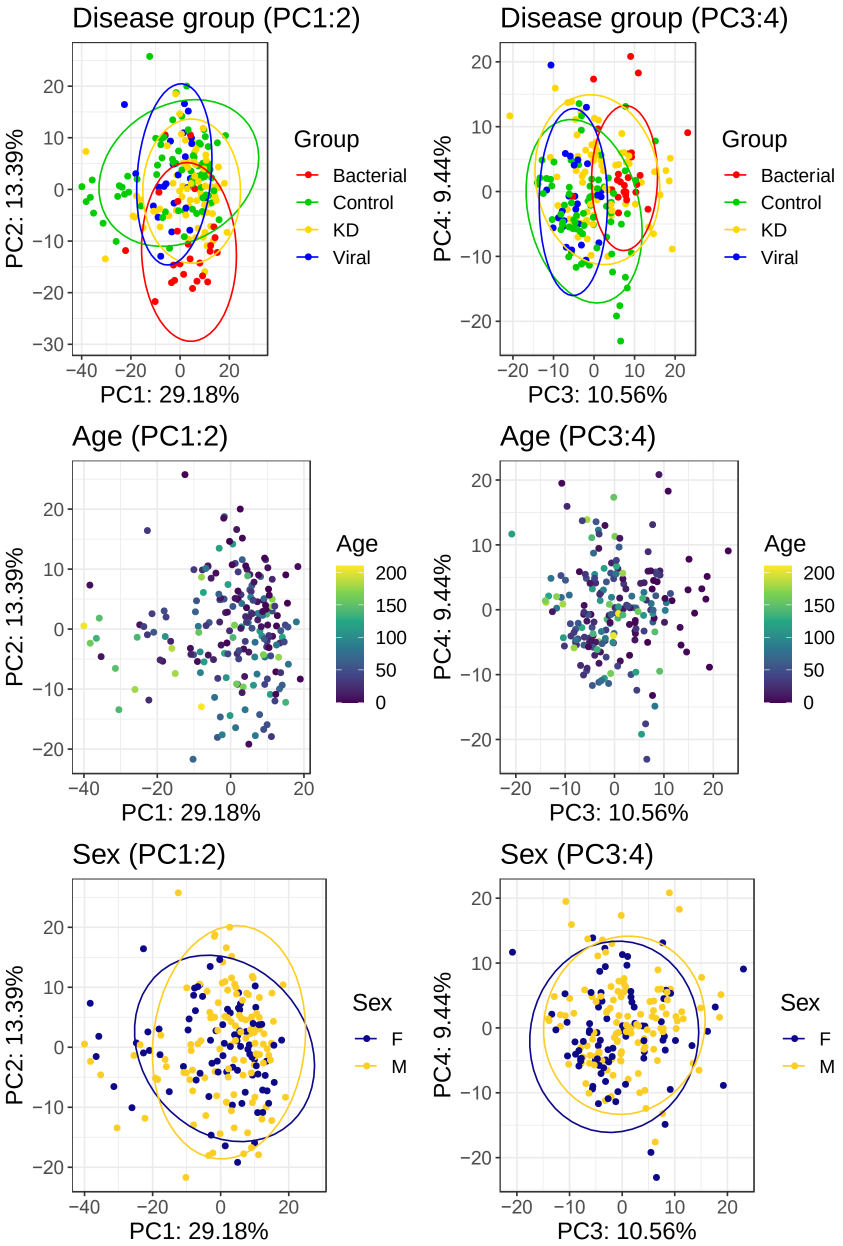

Figure S2. Principal component analysis (PCA) plots for the proteomic dataset with points coloured by disease group, age and sex for principal components 1:4.

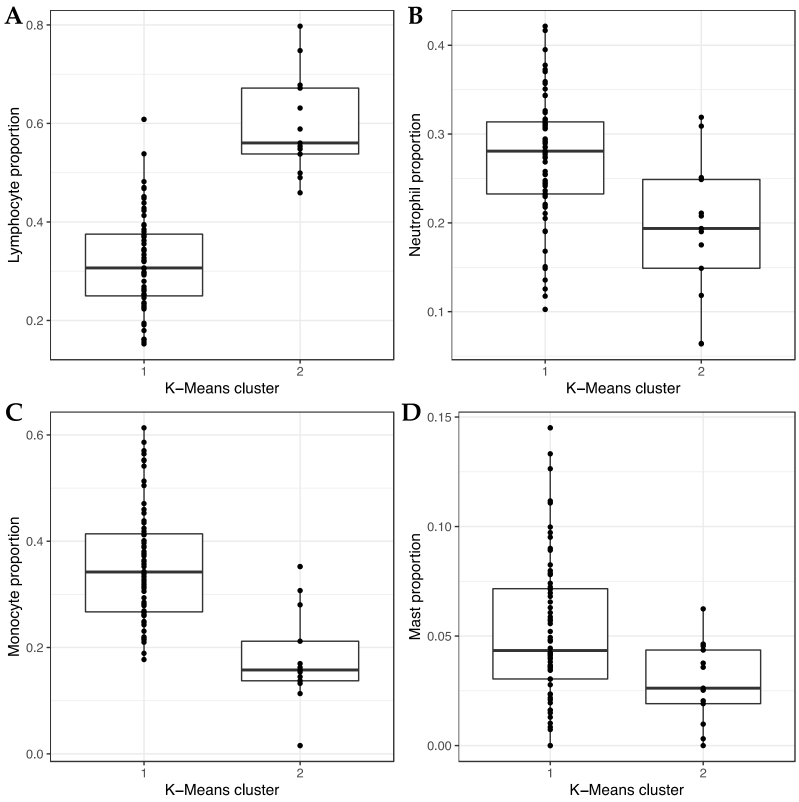

Figure S3. The proportion of lymphocytes (A), neutrophils (B), monocytes (C) and mast cells (D) in transcriptomic KD, bacterial and viral samples for each cluster. Clusters were identified by *K*-Means applied to KD, bacterial and viral samples' expression data without correcting for immune cell proportions

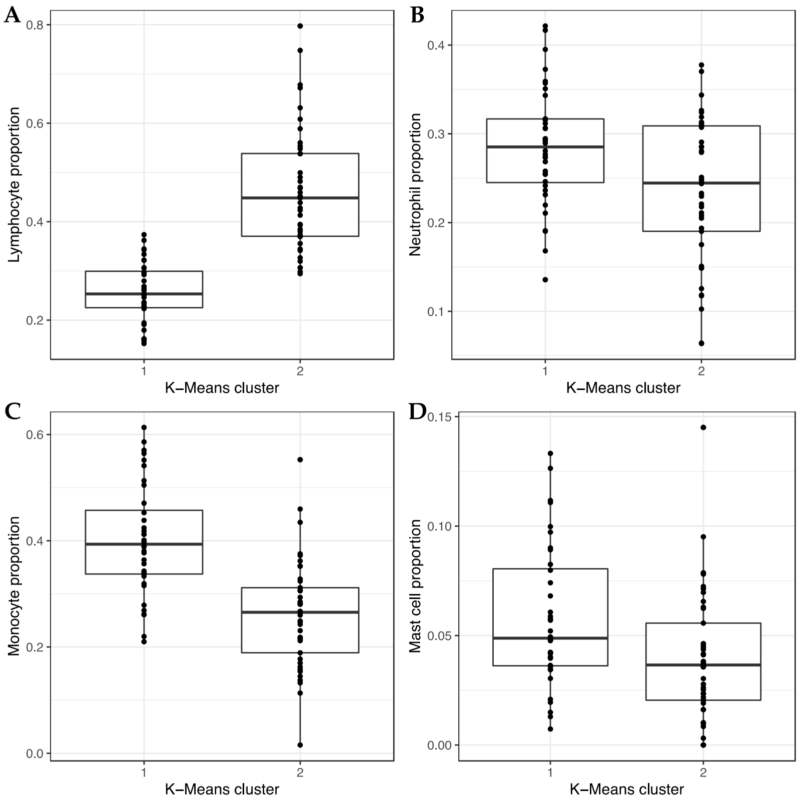

Figure S4. The proportion of lymphocytes (A), neutrophils (B), monocytes (C) and mast cells (D) in transcriptomic KD samples for each cluster. Clusters were identified by *K*-Means applied to KD samples' expression data without correcting for immune cell proportions

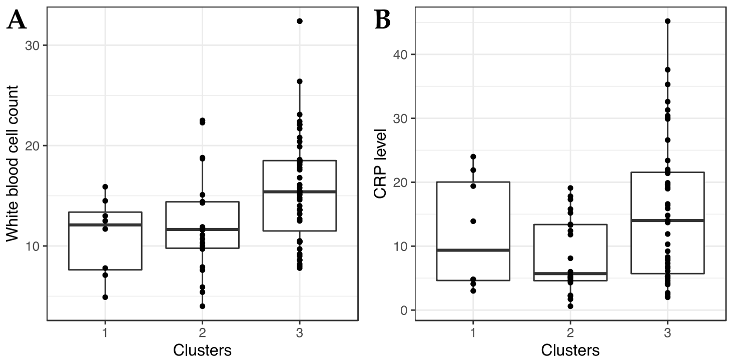

Figure S5. The white blood cell (WBC) counts (A) and CRP levels (B) for KD samples in the 3 clusters identified by *K*-Means clustering when applied to KD, bacterial and viral transcriptomic samples.

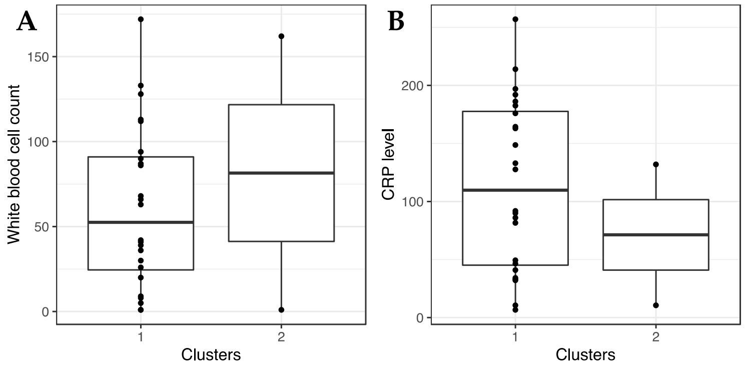

Figure S6. The white blood cell (WBC) counts (A) and CRP levels (B) for KD samples in the 2 clusters identified by *K*-Means clustering when applied to KD, bacterial and viral proteomic samples.

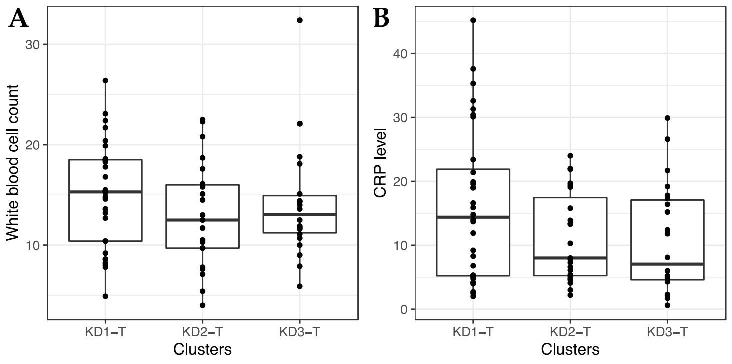

Figure S7. The white blood cell (WBC) count (A) and CRP levels (B) for KD samples in the 3 clusters identified by *K*-Means clustering when applied to KD transcriptomic samples.

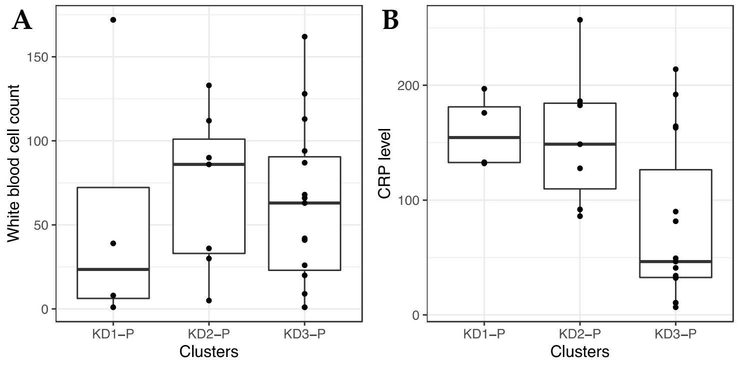

Figure S8. The white blood cell (WBC) count (A) and CRP levels (B) for KD samples in the 3 clusters identified by *K*-Means clustering when applied to KD proteomic samples.

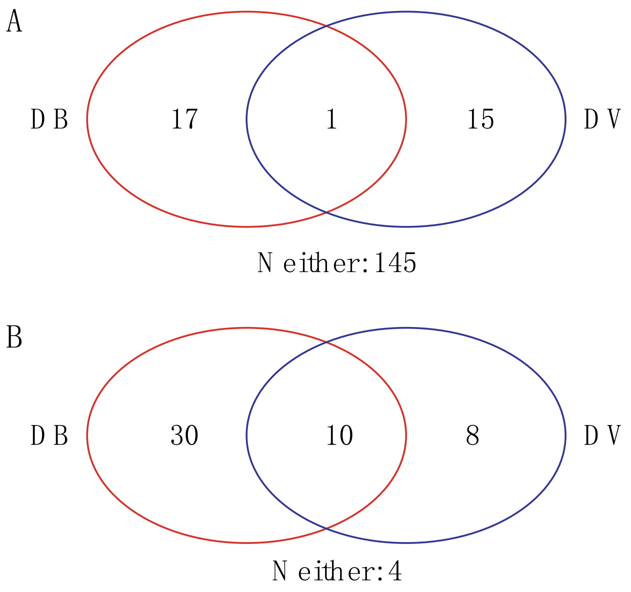

Figure S9. Venn Diagram showing the number of KD patients classified as definite bacterial (DB) or definite viral (DV) (including neither or both DB and DV) in the transcriptomic (A) and proteomic (B) datasets.

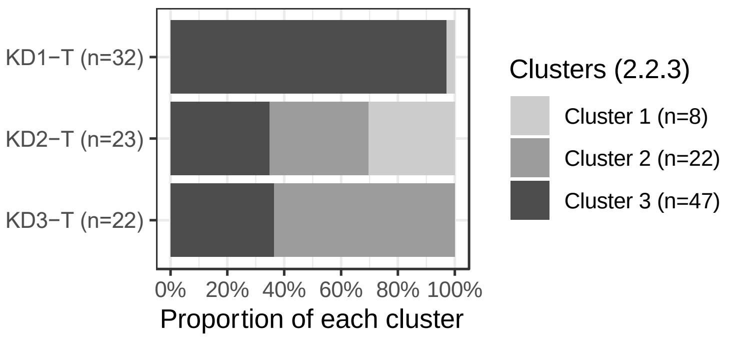

Figure S10. The proportion of KD transcriptomic samples assigned to clusters KD1-T, KD2-T and KD3-T coloured according to the cluster they were assigned previously in *2.2.3*.
