# Supplementary material for: Kawasaki Disease Patient Stratification and Pathway Analysis Based on Host Transcriptomic and Proteomic Profiles
Source: Int J Mol Sci. 2021 May 26;22(11):5655. doi: 10.3390/ijms22115655 (PMC8198135; doi:10.3390/ijms22115655)
Supplement: Supplementary file 1 [file ijms-22-05655-s001.zip › Supplementary Tables.pdf]

Table S1. Patient information for each dataset split by disease group. Ages are in months with age range in brackets.

	Kawasaki disease			Definite bacterial			Definite viral			Healthy controls		
	N	Median age	Males	*N*	Median age	Males	*N*	Median age	Males	*N*	Median age	Males
Transcriptomics												
Discovery	77	26 (3-178)	42 (55%)	31	22 (0-171)	16 (52%)	92	14 (0-175)	65 (70%)	62	40 (0-182)	31 (50%)
Validation	101	37 (4-182)	61 (60%)	23	22 (7-132)	10 (44%)	28	18 (0-172)	17 (60%)	*NA*	*NA*	*NA*
Proteomics												
Discovery	26	30 (3-210)	18 (69%)	73	57 (0-205)	41 (56%)	75	37 (0-180)	42 (56%)	25	58 (1-155)	17 (68%)
Validation	26	16 (0-210)	18 (69%)	48	25 (1-191)	24 (50%)	31	11 (0-97)	14 (45%)	25	101 (3-191)	12 (48%)

Table S2. Clinical information for the KD samples in all four datasets used in the analysis.

	Transcriptomics		*Proteomics*	
	Discovery	Validation	Discovery	Validation
No. of patients	77	101	26	26
Age, median (range), months	26 (3-178)	37 (4-182)	30 (3-210)	16 (0-210)
Male sex, No. (%)	42 (55)	61 (60)	18 (69)	18 (69)
Day of fever at sample collection, median (range)	5 (2 to 7)	6 (2 to 10)	7 (3 to 20)	6.5 (4 to 22)
Coronary artery abnormalities, No (%)	33 (43)	27 (27)	8 (30)	6 (23)
Laboratory values, median (IQR)				
Hemoglobin z-score	-1.3 (-2.0 to -0.3)	-1.2 (-2.0 to -0.4)	-0.22 (-0.24 to -0.19)	-0.3 (-0.31 to -0.26)
C-reactive protein mg/L	119 (48 to 192)	87 (59 to 173)	92 (43 to 167)	92 (59 to 167)
Platelet count x10^3/µl	352 (303 to 448)	408 (324 to 474)	319 (252 to 407)	303 (250 to 425)
White blood cell count /µl	14 200 (10 400 to 18 300)	13 900 (11 000 to 19 000)	13 900 (12 150 to 18 275)	13300 (10 500 to 18 200)
Neutrophil count /µl	9000 (6600 to 12 400)	10 000 (7 300 to 12 600)	10 500 (7125 to 14 200)	8800 (6600 to 11 950)

Table S3 shows the frequency of causative pathogens or disease present in the four datasets used. Causative pathogen refers to the pathogen identified that is causing infection.

Pathogen/disease	Transcriptomic discovery	Transcriptomic validation	Proteomic discovery	Proteomic validation
Bacterial	*n = 31*	*n = 23*	*n = 73*	*n = 48*
Unknown	0	1	4	2
Gram negative bacterial	*n = 14*	*n = 0*	*n = 38*	*n = 23*
Meningococcal	10	0	5	11
E. coli	2	0	21	1
H. influenzae	1	0	3	1
P. aeruginosa	0	0	4	3
C. freundi	0	0	2	0
K. pneumoniae	0	0	1	2
P. mirabilis	0	0	1	0
K. kingae	1	0	0	1
S. marcescens	0	0	1	0
Pertussis	0	0	0	2
Coliform	0	0	0	1
Campylobacter	0	0	0	1
Gram positive bacterial	*n = 17*	*n = 22*	*n = 31*	*n = 23*
Pneumococcus	6	15	3	5
Group A strep.	6	5	14	5
Group B strep.	3	0	0	0
Necrotizing pneumonia	1	0	0	0
S. aureus	1	2	9	8
Enterococcus	0	0	4	1
A. urinae	0	0	1	0
S. anginosus	0	0	0	2
S. warneri	0	0	0	1
Actinomyces	0	0	0	1
Viral	*n = 92*	*n = 28*	*n = 75*	*n = 31*
Unknown	5	1	0	1
RSV	27	10	6	5
Adenovirus	23	2	9	3
Influenza	23	13	19	3
Rhinovirus	6	1	10	4
Bronchiolitis	3	0	0	0
URTI	1	0	0	0
Parainfluenza	1	0	3	0
Parechovirus	1	0	0	3
Pneumonia	1	0	0	0
HSV	1	0	4	1
Measles	0	0	4	0
EBV	0	0	7	0
Enterovirus	0	1	4	6
Coronavirus (not SARS-CoV-2)	0	0	1	0
Cytomegalovirus	0	0	1	0
HMPV	0	0	2	0
Norovirus	0	0	1	0
VZV	0	0	1	0
Rotovirus	0	0	0	2
HHV6	0	0	0	3

Table S4. The genes selected by Lasso in the bacterial classifier with their Lasso weights. Positive Lasso weights indicate higher abundance in bacterial transcriptomic samples compared to viral and healthy control transcriptomic samples and negative Lasso weights indicate lower abundance in bacterial transcriptomic samples.

Illumina ID	Gene symbol	Lasso weight
ILMN_1684271	ACBD6	2.26
ILMN_1779547	HPSE	1.84
ILMN_1662427	PTP4A3	1.77
ILMN_1711368	TRAPPC9	1.21
ILMN_2087692	CYBRD1	1.13
ILMN_1749115	RTN2	0.99
ILMN_2169490	TDRD9	0.78
ILMN_1806612	LOC647104	0.67
ILMN_1663035	SREBF1	0.65
ILMN_3202002	LOC643332	0.61
ILMN_1803819	IQGAP1	0.60
ILMN_1677200	CYFIP2	0.60
ILMN_2058795	PGCP	0.56
ILMN_1807540	CBARA1	0.56
ILMN_1673544	C1orf83	0.54
ILMN_1688780	S100A4	0.53
ILMN_1764769	VWA5A	0.52
ILMN_3295847	LOC645387	0.51
ILMN_1698225	MYO5A	0.50
ILMN_1758672	FAM107B	0.45
ILMN_2397028	SERPINB8	0.42
ILMN_1794967	EIF4ENIF1	0.42
ILMN_2121816	GPR137B	0.27
ILMN_1690342	LTA4H	0.21
ILMN_1814924	FAM55C	0.20
ILMN_3262439	FLJ46552	0.03
ILMN_1664464	PTGDS	-0.09
ILMN_1769299	MTMR11	-0.11
ILMN_1677693	GPR109B	-0.14
ILMN_1699856	RALGDS	-0.27
ILMN_1712026	NLRP3	-0.28
ILMN_1706455	TTC5	-0.40
ILMN_2320336	CLK3	-0.57
ILMN_2310896	NLRP3	-0.74
ILMN_1761828	E2F4	-0.89
ILMN_1785037	SSR2	-1.13
ILMN_1754076	CACNA2D3	-1.20
ILMN_1762594	NOD2	-1.47

Table S5. The genes selected by Lasso in the viral classifier with their Lasso weights. Positive Lasso weights indicate higher abundance in viral transcriptomic samples compared to bacterial and healthy control transcriptomic samples and negative Lasso weights indicate lower abundance in viral transcriptomic samples.

Illumina ID	Gene symbol	Lasso weight
ILMN_2099586	CCDC28B	1.15
ILMN_2348975	NASP	0.88
ILMN_2330307	SLC43A3	0.62
ILMN_2058782	IFI27	0.41
ILMN_1667893	TNS3	0.23
ILMN_1761010	PCCB	0.19
ILMN_1714990	DBT	0.18
ILMN_1751120	HIST1H4H	0.14
ILMN_1747744	LHFPL2	0.05
ILMN_1672124	C4orf18	0.05
ILMN_1811955	PRMT5	0.03
ILMN_3306019	LOC729535	0.01
ILMN_1669062	CCR2	0.01
ILMN_2128795	LRIG1	-0.01
ILMN_2087692	CYBRD1	-0.03
ILMN_1655549	SIGLEC10	-0.03
ILMN_1718936	LST1	-0.03
ILMN_1770824	ARHGAP4	-0.04
ILMN_1864900	MIAT	-0.04
ILMN_2360401	LFNG	-0.04
ILMN_2321578	P2RY10	-0.06
ILMN_1666546	DUSP14	-0.08
ILMN_1738579	VHL	-0.19
ILMN_1803423	ARHGEF6	-0.21
ILMN_1729237	CYB5R1	-0.22
ILMN_1767509	DEF8	-0.24
ILMN_1799030	CMTM2	-0.34
ILMN_1794677	TMC6	-0.45
ILMN_1688373	LST1	-0.50
ILMN_1757827	ECOP	-0.61
ILMN_1712469	GOLGA8A	-0.75
ILMN_2231020	TMEM185B	-1.08

Table S6. The proteins selected by Lasso in the bacterial classifier with their Lasso weights. Positive Lasso weights indicate higher abundance in bacterial samples compared to viral and healthy control samples and negative Lasso weights indicate lower abundance in bacterial samples. Uniprot IDs with * mean that the LFC direction and Lasso direction were not concordant.

Uniprot	Symbol	Lasso weight
P05090*	APOD	25.02
Q14624	ITIH4	22.5
P01011	AACT	15.29
P00740	FA9	10.79
P05543*	THBG	10.62
P01042*	KNG1	9.86
P00751	CFAB	9.39
P0DJI8	SAA1	3.96
P08697*	A2AP	1.85
O14791*	APOL1	1.13
P01031	CO5	0.98
Q96IY4*	CBPB2	0.7
Q9UK55*	ZPI	-0.57
P10643	CO7	-0.93
P02743*	SAMP	-3.58
O00533	NCHL1	-3.83
P02649	APOE	-4.59
O75636	FCN3	-5.06
P12259	FA5	-7.57
P02765	FETUA	-8.54
P48740	MASP1	-10.62
P00747	PLMN	-11.82
P10909	CLUS	-15.19
P04004	VTNC	-20.56
P01008	ANT3	-26.25
P05155	IC1	-27.65

Table S7 The proteins selected by Lasso in the viral classifier with their Lasso weights. Positive Lasso weights indicate higher abundance in viral samples compared to bacterial and healthy control samples and negative Lasso weights indicate lower abundance in viral samples. Uniprot IDs with * mean that the LFC direction and Lasso direction were not concordant.

Uniprot	Symbol	Lasso weight
P05155	IC1	30.93
P04004	VTNC	24.06
P10909	CLUS	16.97
P01008	ANT3	12.98
O75636	FCN3	9.44
P00747	PLMN	5.42
P05543	THBG	3.06
P02765	FETUA	2.48
P02743	SAMP	2.31
Q08380	LG3BP	2.01
O00533	NCHL1	0.05
P01031	CO5	-0.19
P01011	AACT	-0.75
P04196	HRG	-1.57
P05090*	APOD	-1.8
P00751*	CFAB	-2.5
Q96IY4	CBPB2	-3.47
P01042*	KNG1	-8.28
P00740	FA9	-8.98
Q14624	ITIH4	-15.83
