# Supplementary material for: Kawasaki Disease Patient Stratification and Pathway Analysis Based on Host Transcriptomic and Proteomic Profiles
Source: Int J Mol Sci. 2021 May 26;22(11):5655. doi: 10.3390/ijms22115655 (PMC8198135; doi:10.3390/ijms22115655)
Supplement: Supplementary file 1 [file ijms-22-05655-s001.zip › Supplementary Text.pdf]

Supplementary Methods

Case Definitions [1–3]

Kawasaki disease (KD) was diagnosed using American Heart Association (AHA) criteria [4], with 2-dimensional echocardiography performed soon after presentation (2 and 6 weeks after onset). Patients with fewer than 4 of the 5 classic criteria (bilateral nonpurulent conjunctivitis, oral mucosal changes, cervical lymphadenopathy >1.5 cm, peripheral extremity changes and rash) were included as having incomplete KD if the maximum coronary artery z score (Zmax) (standard deviation units from the mean internal diameter normalized for body surface area) at any time during the illness for the left anterior descending or right coronary arteries was 2.5 or higher or if the patients satisfied the algorithm for incomplete KD in the American Heart Association guidelines. Patients were classified as having normal (Zmax <2.5), small (Zmax 2.5 to <5.0), or large (Zmax ≥5.0) CAAs.

Definite bacterial (DB) classification was conditional upon the isolation of a pathogenic bacteria from a sterile site (blood, CSF, pleural space, joint, urine), and a clinical syndrome in keeping with the identified bacterial species. Children diagnosed with other bacterial infections (e.g., mycoplasma, pertussis, mycobacteria) were not included in this group. Identification of bacteria in a sterile-site sample was taken as conclusive evidence for a confirmed bacterial infection, omitting the need for thresholds for inflammatory markers.

Definite viral (DV) classification was conditional upon the identification of a virus matching the syndrome and no evidence of bacterial coinfection. To avoid inclusion of children with occult bacterial infection, thresholds of inflammatory markers were used. C-reactive protein (CRP) levels ≤60mg/L and neutrophil counts ≤12 x 10^9/L were required for DV classification.

Generation of mass spectrometry proteomic dataset

Prior to proteomic quantification using LC-MS/MS in the discovery dataset, a top 12 abundant protein depletion spin column (Catlog number 85165, ThermoFisher Scientific) was used to remove 12 high abundance proteins: α1-acid glycoprotein, α1-antitrypsin, α2-macroglobulin, albumin, apolipoprotein A-I, apolipoprotein A-II, fibrinogen, haptoglobin, IgA, IgG, IgM and transferrin. Ten microliters of plasma were added into each depletion column. Immunodepletion cartridges were rotated at 20 rpm for 1 hour to maxmise immunoaffinity binding. The flow-through was collected by spinning the samples at 1000 *g* for 1 minute. To concentrate proteins in flow through, trichloroacetic acid (TCA) protein precipatation was followed by adding 60 µl of 100% TCA into the T12-abundant depleted samples. Six microliters of 2% sodium deoxycholate (DOC) were added into TCA samples and kept at -80 °C for 1 hour. Samples were centrifuged at 13000 *g* for 30 minutes at 4 °C and the supernatants were discarded. Eight hundred microliters of cold acetone (100%) was added to each sample, incubated at -20 °C for 1 hour and centrifuged at 13000 *g* for 30 minutes. The protein pellet samples were resuspended in 60 µl of 4M urea (pH 7.4 mM Tris). For total protein quantification of the depleted samples, bicinchoninic acid (BCA) protein assays was used (ThermoFisher Scientific). Following protein quantification, the proteins were digested at 70° (denaturing condition) by thermo stable trypsin using the SMART digestion kit (ThermoFisher Scientific). After peptide clean up, 0.5 µg of peptides were injected into a LC-MS/MS (LUMOS Fusion, Thermo Fisher scientific) at the Oxford proteomic facility. Samples were ordered according to disease group (bacterial, viral groups were run ahead of KD group). To avoid bias by system error, the performance of LC-MS/MS (sensitivity of detection, retention time shifting, number of proteins identified) were monitored by running a quality control sample (pool of all individual samples) every 10 samples within batch. A blank in between runs was included cross entire batch which cleans the column and avoid sample carry over.

The raw dataset files were processed by MaxQuant (1.6.10.43) [5] based on default settings with matching between runs activated (20 minutes tolerance) and contaminants included. The FDR cut-off for proteins and peptides was 1% with razor protein FDR enabled and the variable modifications were methionine oxidation and protein N-terminal acetylation. Maximum peptide

mass was 4600 Da. Second peptide search was enabled. Relative quantification was performed using the MaxLFQ algorithm [6].

Supplementary Results

Differential abundance analysis results

Supplementary File 1 (S1_KD_vs_HC_Features.xlsx) contains the genes and proteins significantly differentially abundant between KD and healthy controls

Pathway analysis results

Supplementary Files 2-5 all contain uniqueness and dispensability metrics for pathway analysis. These are both terms returned by REVIGO [7] that quantify the extent to which a GO term is an outlier compared to the rest of the group (uniqueness), and the similarity of a GO term to other semantically close terms (dispensability).

Supplementary File 2 (S2_Pathways_Disease_Groups.xlsx) contains the pathways upregulated or downregulated when comparing KD vs healthy controls, definite bacterial vs healthy controls and definite viral vs healthy controls.

Supplementary File 3 (S3_Pathways_Clusters_Transcriptomics.xlsx) contains the pathways upregulated or downregulated when comparing the KD patients within the 3 different clusters identified in the transcriptomic dataset through *K*-Means clustering applied to KD, bacterial and viral patients.

Supplementary File 4 (S4_Pathways_KD_Clusters_Transcriptomics.xlsx) shows the pathways upregulated or downregulated when comparing the KD patients within the 3 different clusters identified in the transcriptomic dataset through *K*-Means clustering applied to KD patients alone.

Supplementary File 5 (S5_Pathways_KD_Clusters_Proteomics.xlsx) shows the pathways upregulated or downregulated when comparing the KD patients within the 3 different clusters identified in the proteomic dataset through *K*-Means clustering applied to KD patients alone.

K-Means clustering

K-Means clustering was applied to KD, bacterial and viral samples, and separately to KD samples alone. When KD, bacterial and viral transcriptomic samples were subjected to *K*-Means clustering without having removed the contribution of immune cell proportions, two clusters were optimal, and these clusters corresponded strongly to immune cell proportions (Fig. S3). Cluster 1 contained patients with low lymphocyte proportions and high neutrophil, mast cell and monocyte proportions. Cluster 2 contained patients with high lymphocyte proportions and low neutrophil, mast cell and monocyte proportions. The same pattern was observed when *K*-Means clustering was applied to the KD samples alone where 2 clusters was also optimal (Fig. S4).

When clustering was applied to the corrected data for KD, bacterial and viral transcriptomic samples, 3 clusters were optimal. When clustering was applied to the KD proteomic samples, 2 clusters were optimal. WBC counts and CRP levels were highest for transcriptomic KD samples in cluster 3 (Fig. S5), in which the majority of the bacterial samples was found. Since there were only 2 KD proteomic samples in cluster 2, it is difficult to make comparisons between the clusters, however both KD samples in this cluster, which contained the majority of the viral samples, have lower CRP (Fig. S6).

When clustering was applied to the corrected data for KD patients alone, 3 clusters were optimal on both 'omic levels. For the transcriptomics, CRP levels and, to a lesser extent, WBC counts were highest in cluster KD1-T (Fig. S7), in which various viral-associated pathways were also down regulated. Given the small number of KD samples in some of the proteomic clusters (Fig. S8), it is harder to compare the levels of CRP and WBC counts between clusters. The levels of CRP appear to be slightly higher in cluster KD1-P, in which an enrichment of pathways previously identified in proteomic bacterial samples was observed. The highest WBC counts were observed in cluster KD2-P.

Classifier

Two independent classifiers were built that return the probability of a patient being viral or bacterial. Lasso regularised regression [8] was used to identify the discriminatory features for each classifier. The abundance of these features was used to calculate a disease risk score (DRS). Only features with concordant LFC and Lasso weight direction were used to calculate the DRS.

Of the 38 genes selected by Lasso for the transcriptomic bacterial classifier, 26 have increased abundance in bacterial patients compared to viral and healthy controls, and 12 have decreased abundance in bacterial patients (Table S4). All genes had concordant LFC and Lasso weight directions, therefore all genes listed in table S4 were used to calculate the bacterial DRS (DB-DRS) in the transcriptomic dataset.

Of the 32 genes selected by Lasso for the transcriptomic viral classifier, 13 have increased abundance in viral patients compared to bacterial and healthy controls, and 19 have decreased abundance in bacterial patients (Table S5). All genes had concordant LFC and Lasso weight directions, therefore all genes listed in table S5 were used to calculate the viral DRS (DV-DRS) in the transcriptomic dataset. Only one gene was selected in both classifiers: Cytochrome b reductase (CYBRD1) which has increased abundance in bacterial patients.

Of the 26 proteins selected by Lasso for the proteomic bacterial classifier, 12 have increased abundance in bacterial patients compared to viral and healthy controls, and 14 have decreased abundance in bacterial patients (Table S6). 8 proteins (indicated in table S6 by * next to Uniprot IDs) had discordant LFC and Lasso weight directions. These proteins were not used to calculate the DRS.

Of the 20 proteins selected by Lasso for the proteomic viral classifier, 11 have increased abundance in viral patients compared to bacterial and healthy controls, and 9 have decreased abundance in viral patients (Table S7). 3 proteins (indicated in table S7 by * next to Uniprot IDs) had discordant LFC and Lasso weight directions. These proteins were not used to calculate the DRS.

15 proteins were selected by both Lasso regression models, 6 of which had higher abundance in bacterial patients (FA9, AACT, CO5, KNG1, APOD, ITIH4, CBPB2) and 9 of which had higher abundance in viral patients (NCHL1, FCN3, PLMN, ANT3, SAMP, FETUA, VTNC, IC1, CLUS). One protein, THBG, had positive Lasso weights in both models, indicating that its abundance was increased in both bacterial and viral samples. This protein was retained for the viral classifier as its LFC and Lasso weight directions were concordant, unlike in the bacterial classifier. There were no features for which both of the protein and gene pair were selected.

Figure S9 shows the number of KD samples classified as bacterial or viral, both, or neither for the transcriptomic (A) and proteomic (B) datasets.

Supplementary Information

PERFORM consortium author list

PARTNER: IMPERIAL COLLEGE (UK)

Chief investigator/PERFORM coordinator:

Michael Levin

Principal and co-investigators; work package leads (alphabetical order)

Aubrey Cunnington (grant application)

Tisham De (work package lead)

Jethro Herberg (Principle Investigator, Deputy Coordinator, grant application)

Myrsini Kaforou (grant application, work package lead)

Victoria Wright (grant application, Scientific Coordinator)

Research Group (alphabetical order)
Lucas Baumard; Evangelos Bellos; Giselle D'Souza; Rachel Galassini; Dominic Habgood-Coote; Shea Hamilton; Clive Hoggart; Sara Hourmat; Heather Jackson; Ian Maconochie; Stephanie Menikou; Naomi Lin; Samuel Nichols; Ruud Nijman; Ivonne Pena Paz; Priyen Shah; Ching-Fen Shen; Clare Wilson

Clinical recruitment at Imperial College Healthcare NHS Trust (alphabetical order)
Amina Abdulla; Ladan Ali; Sarah Darnell; Rikke Jorgensen; Sobia Mustafa; Salina Persand

Imperial College Faculty of Engineering
Molly Stevens (co-investigator), Eunjung Kim (research group); Benjamin Pierce (research group)

Clinical recruitment at Brighton and Sussex University Hospitals
Katy Fidler (Principle Investigator)
Julia Dudley (Clinical Research Registrar)
Research nurses: Vivien Richmond, Emma Tavliavini

Clinical recruitment at National Cheng Kung University Hospital
Ching-Fen Shen (Principal Investigator); Ching-Chuan Liu (Co-investigator); Shih-Min Wang (Co-investigator), funded by the Center of Clinical Medicine Research, National Cheng Kung University

PARTNER: SERGAS (Spain)
Principal Investigators
Federico Martinón-Torres[1]
Antonio Salas[1,2]

Research Group (alphabetical order)
Fernando Álvez González[1], Cristina Balo Farto[1], Ruth Barral-Arca[1,2], María Barreiro Castro[1], Xabier Bello[1,2], Mirian Ben García[1], Sandra Carnota[1], Miriam Cebey-López[1], María José Curras-Tuala[1,2], Carlos Durán Suárez[1], Luisa García Vicente[1], Alberto Gómez-Carballa[1,2], Jose Gómez Rial[1], Pilar Leboráns Iglesias[1], Federico Martinón-Torres[1], Nazareth Martinón-Torres[1], José María Martinón Sánchez[1], Belén Mosquera Pérez[1], Jacobo Pardo-Seco[1,2], Lidia Piñeiro Rodríguez[1], Sara Pischedda[1,2], Sara Rey Vázquez[1], Irene Rivero Calle[1], Carmen Rodríguez-Tenreiro[1], Lorenzo Redondo-Collazo[1], Miguel Sadiki Ora[1], Antonio Salas[1,2], Sonia Serén Fernández[1], Cristina Serén Trasorras[1], Marisol Vilas Iglesias[1].

[1] Translational Pediatrics and Infectious Diseases, Pediatrics Department, Hospital Clínico Universitario de Santiago, Santiago de Compostela, Spain, and GENVIP Research Group (www.genvip.org), Instituto de Investigación Sanitaria de Santiago, Universidad de Santiago de Compostela, Galicia, Spain.
[2] Unidade de Xenética, Departamento de Anatomía Patolóxica e Ciencias Forenses, Instituto de Ciencias Forenses, Facultade de Medicina, Universidade de Santiago de Compostela, and GenPop Research Group, Instituto de Investigaciones Sanitarias (IDIS), Hospital Clínico Universitario de Santiago, Galicia, Spain
[3] Fundación Pública Galega de Medicina Xenómica, Servizo Galego de Saúde (SERGAS), Instituto de Investigaciones Sanitarias (IDIS), and Grupo de Medicina Xenómica, Centro de Investigación Biomédica en Red de Enfermedades Raras (CIBERER), Universidade de Santiago de Compostela (USC), Santiago de Compostela, Spain

PARTNER: RSU (Latvia)

Principal Investigator
Dace Zavadska[1,2]

Other RSU group authors (in alphabetical order):
Anda Balode[1,2], Arta Bārzdiņa[1,2], Dārta Deksne[1,2], Dace Gardovska[1,2], Dagne Grāvele[2], Ilze Grope[1,2], Anija Meiere[1,2], Ieva Nokalna[1,2], Jana Pavāre[1,2], Zanda Pučuka[1,2], Katrīna Selecka[1,2], Aleksandra Sidorova[1,2], Dace Svile[2], Urzula Nora Urbāne[1,2].

[1] Riga Stradins university, Riga, Latvia.
[2] Children clinical university hospital, Riga, Latvia.

PARTNER: Medical Research Council Unit The Gambia (MRCG) at LSHTM

Principal Investigator
Effua Usuf

Additional Investigators
Kalifa Bojang
Syed M. A. Zaman
Fatou Secka
Suzanne Anderson
Anna RocaIsatou Sarr
Momodou Saidykhan
Saffiatou Darboe
Samba Ceesay
Umberto D'alessandro

Medical Research Council Unit The Gambia at LSHTM
P O Box 273,
Fajara, The Gambia

PARTNER: ERASMUS MC-Sophia Children's Hospital (Netherlands)

Principal Investigator
Henriëtte A. Moll[1]

Research Group (alphabetical order)
Dorine M. Borensztajn[1], Nienke N. Hagedoorn, Chantal Tan [1], [1], Clementien L. Vermont[2], Joany Zachariasse [1]

Additional investigator
W Dik [3]

[1] Erasmus MC-Sophia Children's Hospital, Department of General Paediatrics, Rotterdam, the Netherlands
[2] Erasmus MC-Sophia Children's Hospital, Department of Paediatric Infectious Diseases & Immunology, Rotterdam, the Netherlands
[3] Erasmus MC, Department of immunology, Rotterdam, the Netherlands

PARTNER: Swiss Pediatric Sepsis Study (Switzerland)

Principal Investigators:
Philipp Agyeman, MD [1] (ORCID 0000-0002-8339-5444), Luregn J Schlapbach, MD, FCICM [2,3] (ORCID 0000-0003-2281-2598)

Clinical recruitment at University Children's Hospital Bern for PERFORM:
Christoph Aebi [1], Verena Wyss [1], Mariama Usman [1]

Principal and co-investigators for the Swiss Pediatric Sepsis Study:
Philipp Agyeman, MD [1], Luregn J Schlapbach, MD, FCICM [2,3], Eric Giannoni, MD [4,5], Martin Stocker, MD [6], Klara M Posfay-Barbe, MD [7], Ulrich Heininger, MD [8], Sara Bernhard-Stirnemann, MD [9], Anita Niederer-Loher, MD [10], Christian Kahlert, MD [10], Giancarlo Natalucci, MD [11], Christa Relly, MD [12], Thomas Riedel, MD [13], Christoph Aebi, MD [1], Christoph Berger, MD [12] for the Swiss Pediatric Sepsis Study

[1] Department of Pediatrics, Inselspital, Bern University Hospital, University of Bern, Switzerland
[2] Neonatal and Pediatric Intensive Care Unit, Children's Research Center, University Children's Hospital Zurich, University of Zurich, Zurich, Switzerland
[3]Child Health Research Centre, University of Queensland, and Queensland Children`s Hospital, Brisbane, Australia
[4] Clinic of Neonatology, Department Mother-Woman-Child, Lausanne University Hospital and University of Lausanne, Switzerland
[5] Infectious Diseases Service, Department of Medicine, Lausanne University Hospital and University of Lausanne, Switzerland
[6] Department of Pediatrics, Children's Hospital Lucerne, Lucerne, Switzerland
[7] Pediatric Infectious Diseases Unit, Children's Hospital of Geneva, University Hospitals of Geneva, Geneva, Switzerland
[8] Infectious Diseases and Vaccinology, University of Basel Children's Hospital, Basel, Switzerland
[9] Children's Hospital Aarau, Aarau, Switzerland
[10] Division of Infectious Diseases and Hospital Epidemiology, Children's Hospital of Eastern Switzerland St. Gallen, St. Gallen, Switzerland
[11] Department of Neonatology, University Hospital Zurich, Zurich, Switzerland
[12] Division of Infectious Diseases and Hospital Epidemiology, and Children's Research Center, University Children's Hospital Zurich, Switzerland
[13] Children's Hospital Chur, Chur, Switzerland

PARTNER: Liverpool (UK)

Principal Investigators
Enitan D Carrol[1,2,3]
Stéphane Paulus [1,]

Research Group (alphabetical order)
Elizabeth Cocklin[1], Rebecca Jennings[4], Joanne Johnston[4], Simon Leigh[1], Karen Newall[4], Sam Romaine[1]

[1] Department of Clinical Infection, Microbiology and Immunology, University of Liverpool Institute of Infection and Global Health , Liverpool, England
[2] Alder Hey Children's Hospital, Department of Infectious Diseases, Eaton Road, Liverpool, L12 2AP
[3] Liverpool Health Partners, 1st Floor, Liverpool Science Park, 131 Mount Pleasant, Liverpool, L3 5TF
[4]Alder Hey Children's Hospital, Clinical Research Business Unit, Eaton Road, Liverpool, L12 2AP

PARTNER: NKUA (Greece)

Principal investigator
Professor Maria Tsolia (all activities)

Investigator/Research fellow
Irini Eleftheriou (all activities)

Additional investigators
Recruitment: Maria Tambouratzi
Lab: Antonis Marmarinos (Quality Manager)
Lab: Marietta Xagorari
Kelly Syggelou
2nd Department of Pediatrics, National and Kapodistrian University of Athens,
"P. and A. Kyriakou" Children's Hospital
Thivon and Levadias
Goudi, Athens

PARTNER: Micropathology Ltd (UK)

Principal Investigator
Professor Colin Fink[1], Clinical Microbiologist

Additional investigators
Dr Marie Voice[1], Post doc scientist
Dr. Leo Calvo-Bado[1], Post doc scientist
[1] Micropathology Ltd, The Venture Center, University of Warwick Science Park, Sir William Lyons Road, Coventry, CV4 7EZ.

PARTNER: Medical University of Graz (MUG, Austria)

Principal Investigator
Werner Zenz[1] (all activities)

Co-investigators (alphabetical order)
Benno Kohlmaier[1] (all activities)
Nina A. Schweintzger[1] (all activities)
Manfred G. Sagmeister[1] (study design, consortium wide sample management)
Research team
Daniela S. Kohlfürst[1] (study design)
Christoph Zurl[1] (BIVA PIC)
Alexander Binder[1] (grant application)

Recruitment team, data managers, (alphabetical order)
Susanne Hösele[1], Manuel Leitner[1], Lena Pölz[1], Glorija Rajic[1],

Clinical recruitment partners (alphabetical order)
Sebastian Bauchinger[1], Hinrich Baumgart[4], Martin Benesch[3], Astrid Ceolotto[1], Ernst Eber[2], Siegfried Gallistl[1], Gunther Gores[5], Harald Haidl[1], Almuthe Hauer[1], Christa Hude[1], Markus Keldorfer[5], Larissa Krenn[4], Heidemarie Pilch[5], Andreas Pfleger[2], Klaus Pfurtscheller[4], Gudrun Nordberg[5], Tobias

Niedrist[8], Siegfried Rödl[4], Andrea Skrabl-Baumgartner[1], Matthias Sperl[7], Laura Stampfer[5], Volker Strenger[3], Holger Till[6], Andreas Trobisch[5], Sabine Löffler[5]

[1] Department of Pediatrics and Adolescent Medicine, Division of General Pediatrics, Medical University of Graz, Graz, Austria
[2]Department of Pediatric Pulmonology, Medical University of Graz, Graz, Austria
[3]Department of Pediatric Hematooncoloy, Medical University of Graz, Graz, Austria
[4]Paediatric Intensive Care Unit, Medical University of Graz, Graz, Austria
[5]University Clinic of Paediatrics and Adolescent Medicine Graz, Medical University Graz, Graz,Austria
[6]Department of Paediatric and Adolescence Surgery, Medical University Graz, Graz, Austria
[7]Department of Pediatric Orthopedics, Medical University Graz, Graz, Austria
[8]Clinical Institute of Medical and Chemical Laboratory Diagnostics, Medical University Graz, Graz, Austria

PARTNER: London School of Hygiene and Tropical Medicine (UK)

WP 1 WP2, WP5
Principal Investigator:
Dr Shunmay Yeung[1,2 3] PhD, MBBS, FRCPCH, MRCP, DTM&H

Research Group
Dr Juan Emmanuel Dewez[1] MD, DTM&H, MSc
Prof Martin Hibberd [1] BSc, PhD
Mr David Bath[2] MSc, MAppFin, BA(Hons)
Dr Alec Miners[2] BA(Hons), MSc, PhD
Dr Ruud Nijman[3] PhD MSc MD MRCPCH
Dr Catherine Wedderburn[1] BA, MBChB, DTM&H, MSc, MRCPCH
Ms Anne Meierford[1] MSc, BMedSc, BMBS
Dr Baptiste Leurent[4], PhD, MSc

1. Faculty of Infectious and Tropical Disease, London School of Hygiene and Tropical Medicine, London, UK
2. Faculty of Public Health and Policy, London School of Hygiene and Tropical Medicine, London, UK
3. Department of Paediatrics, St. Mary's Hospital Imperial College Hospital, London, UK
4. Faculty of Epidemiology and Population Health, London School of Hygiene and Tropical Medicine, London, UK

PARTNER: Radboud University Medical Center (RUMC, Netherlands)

Principal Investigators
Ronald de Groot [1], Michiel van der Flier [1,2,3], Marien I. de Jonge[1]

Co-investigators Radboud University Medical Center (alphabetical order)
Koen van Aerde[1,2], Wynand Alkema[1], Bryan van den Broek[1], Jolein Gloerich[1], Alain J. van Gool[1], Stefanie Henriet[1,2], Martijn Huijnen[1], Ria Philipsen[1], Esther Willems[1]

Investigators PeDBIG PERFORM DUTCH CLINICAL NETWORK (alphabetical order)
G.P.J.M. Gerrits[8], M. van Leur[8,] J. Heidema [4],L. de Haan[1,2] C.J. Miedema [5], C. Neeleman [1] C.C. Obihara [6], G.A. Tramper-Stranders7[6]

1. Radboud University Medical Center, Nijmegen, The Netherlands
2. Amalia Children's Hospital, Nijmegen, The Netherlands
3. Wilhelmina Children's Hospital, University Medical Center Utrecht, Utrecht, The Netherlands
4. St. Antonius Hospital, Nieuwegein, The Netherlands
5. Catharina Hospital, Eindhoven, The Netherlands
6. ETZ Elisabeth, Tilburg, The Netherlands
7. Franciscus Gasthuis, Rotterdam, The Netherlands
8. Canisius Wilhelmina Hospital, Nijmegen, The Netherlands

PARTNER: Oxford (UK)

Principal Investigators
Andrew J. Pollard[1,2], Rama Kandasamy[1,2], Stéphane Paulus [1,2]

Additional Investigators
Michael J. Carter[1,2], Daniel O'Connor[1,2], Sagida Bibi[1,2], Dominic F. Kelly[1,2], Meeru Gurung[3], Stephen Thorson[3], Imran Ansari[3], David R. Murdoch[4], Shrijana Shrestha[3].

[1]Oxford Vaccine Group, Department of Paediatrics, University of Oxford, Oxford, United Kingdom.
[2]NIHR Oxford Biomedical Research Centre, Oxford, United Kingdom.
[3]Paediatric Research Unit, Patan Academy of Health Sciences, Kathmandu, Nepal.
[4]Department of Pathology, University of Otago, Christchurch, New Zealand.

PARTNER: Newcastle University, Newcastle upon Tyne, (UK)

Principal Investigator
Marieke Emonts [1,2,3] (all activities)

Co-investigators
Emma Lim[2,3,7] (all activities)
Lucille Valentine[4]

Recruitment team (alphabetical), data-managers, and GNCH Research unit
Karen Allen[5], Kathryn Bell[5], Adora Chan[5], Stephen Crulley[5], Kirsty Devine[5], Daniel Fabian[5], Sharon King[5], Paul McAlinden[5], Sam McDonald[5], Anne McDonnell2,[5], Ailsa Pickering[2,5], Evelyn Thomson[5], Amanda Wood[5], Diane Wallia[5], Phil Woodsford[5],
Sample processing: Frances Baxter[5], Ashley Bell[5], Mathew Rhodes[5]

PICU recruitment
Rachel Agbeko[8]
Christine Mackerness[8]

Students MOFICHE
Bryan Baas[2], Lieke Kloosterhuis[2], Wilma Oosthoek[2]

Students/medical staff PERFORM
Tasnim Arif[6], Joshua Bennet[2], Kalvin Collings[2], Ilona van der Giessen[2], Alex Martin[2], Aqeela Rashid[6], Emily Rowlands[2], Gabriella de Vries[2], Fabian van der Velden[2]

Engagement work/ethics/cost effectiveness
Lucille Valentine [4], Mike Martin[9], Ravi Mistry[2], Lucille Valentine[4]

[1] Translational and Clinical Research Institute, Newcastle University, Newcastle upon Tyne UK
[2]Great North Children's Hospital, Paediatric Immunology, Infectious Diseases & Allergy, Newcastle upon Tyne Hospitals NHS Foundation Trust, Newcastle upon Tyne, United Kingdom.
[3]NIHR Newcastle Biomedical Research Centre based at Newcastle upon Tyne Hospitals NHS Trust and Newcastle University, Westgate Rd, Newcastle upon Tyne NE4 5PL, United Kingdom
[4]Newcastle University Business School, Centre for Knowledge, Innovation, Technology and Enterprise (KITE), Newcastle upon Tyne, United Kingdom
[5]Great North Children's Hospital, Research Unit, Newcastle upon Tyne Hospitals NHS Foundation Trust, Newcastle upon Tyne, United Kingdom.
[6]Great North Children's Hospital, Paediatric Oncology, Newcastle upon Tyne Hospitals NHS Foundation Trust, Newcastle upon Tyne, United Kingdom.
[7]Population Health Sciences Institute, Newcastle University, Newcastle upon Tyne, UK
[8]Great North Children's Hospital, Paediatric Intensive Care Unit, Newcastle upon Tyne Hospitals NHS Foundation Trust, Newcastle upon Tyne, United Kingdom.
[9]Northumbria University, Newcastle upon Tyne, United Kingdom.

PARTNER: LMU Munich (Germany)

Principal Investigator
Ulrich von Both[1,2] MD, FRCPCH (all activities)

Research group
Laura Kolberg[1] MSc (all activities)
Manuela Zwerenz[1] MSc, Judith Buschbeck[1] PhD

Clinical recruitment partners (alphabetical order)
Christoph Bidlingmaier[3], Vera Binder[4], Katharina Danhauser[5], Nikolaus Haas[10], Matthias Griese[6], Tobias Feuchtinger[4], Julia Keil[9], Matthias Kappler[6], Eberhard Lurz[7], Georg Muench[8], Karl Reiter[9], Carola Schoen[9]

[1]Div. Paediatric Infectious Diseases, Hauner Children's Hospital, University Hospital, Ludwig Maximilians University (LMU), Munich, Germany
[2]German Center for Infection Research (DZIF), Partner Site Munich, Munich, Germany
[3]Div. of General Paediatrics, [4]Div. Paediatric Haematology & Oncology, [5]Div. of Paediatric Rheumatology, [6]Div. of Paediatric Pulmonology, [7]Div. of Paediatric Gastroenterology, [8]Neonatal Intensive Care Unit, [9]Paediatric Intensive Care Unit Hauner Children's Hospital, University Hospital, Ludwig Maximilians University (LMU), Munich, Germany, [10]Department Pediatric Cardiology and Pediatric Intensive Care, University Hospital, Ludwig Maximilians University (LMU), Munich, Germany

PARTNER: bioMérieux (France)

Principal Investigator
François Mallet[1,2, 3]

Research Group
Karen Brengel-Pesce[1,2, 3]

Alexandre Pachot[1]
Marine Mommert[1,2]

[1]Open Innovation & Partnerships (OIP), bioMérieux S.A., Marcy l'Etoile, France
[2] Joint research unit Hospice Civils de Lyon - bioMérieux, Centre Hospitalier Lyon Sud, 165 Chemin du Grand Revoyet, 69310 Pierre-Bénite, France
[3] EA 7426 Pathophysiology of Injury-induced Immunosuppression, University of Lyon1-Hospices Civils de Lyon-bioMérieux, Hôpital Edouard Herriot, 5 Place d'Arsonval, 69437 Lyon Cedex 3, France

PARTNER: University Medical Centre Ljubljana (Slovenia)

Principal Investigator
Marko Pokorn[1,2,3] MD, PhD

Research Group
Mojca Kolnik[1] MD, Katarina Vincek[1] MD, Tina Plankar Srovin[1] MD, PhD, Natalija Bahovec[1] MD, Petra Prunk[1] MD, Veronika Osterman[1] MD, Tanja Avramoska[1] MD

[1]Department of Infectious Diseases, University Medical Centre Ljubljana, Japljeva 2, SI-1525 Ljubljana, Slovenia
[2]University Childrens' Hospital, University Medical Centre Ljubljana, Ljubljana, Slovenia
[3]Department of Infectious Diseases and Epidemiology, Faculty of Medicine, University of Ljubljana, Slovenia

PARTNER: Amsterdam, Academic Medical Hospital & Sanquin Research Institute (Netherlands)

Principal Investigator
Taco Kuijpers [1,2]

Co-investigators
Ilse Jongerius [2]

Recruitment team (EUCLIDS, PERFORM)
J.M. van den Berg[1], D. Schonenberg[1], A.M. Barendregt[1], D. Pajkrt[1], M. van der Kuip[1,3], A.M. van Furth[1,3]

Students PERFORM
Evelien Sprenkeler [2], Judith Zandstra [2]

Technical support PERFORM
G. van Mierlo [2], J. Geissler [2]

[1] Amsterdam University Medical Center (Amsterdam UMC), location Academic Medical Center (AMC), Dept of Pediatric Immunology, Rheumatology and Infectious Diseases, University of Amsterdam, Amsterdam, the Netherlands
[2] Sanquin Research Institute, & Landsteiner Laboratory at the AMC, University of Amsterdam, Amsterdam, the Netherlands.
[3] Amsterdam University Medical Center (Amsterdam UMC), location Vrije Universiteit Medical Center (VUMC), Dept of Pediatric Infectious Diseases and Immunology, Free University (VU), Amsterdam, the Netherlands (former affiliation)

References

1. Wright, V.J.; Herberg, J.A.; Kaforou, M.; Shimizu, C.; Eleftherohorinou, H.; Shailes, H.; Barendregt, A.M.; Menikou, S.; Gormley, S.; Berk, M.; et al. Diagnosis of Kawasaki Disease Using a Minimal Whole-Blood Gene Expression Signature. *JAMA Pediatr.* **2018**, *172*, doi:10.1001/jamapediatrics.2018.2293.
2. Hoang, L.T.; Shimizu, C.; Ling, L.; Naim, A.N.M.; Khor, C.C.; Tremoulet, A.H.; Wright, V.; Levin, M.; Hibberd, M.L.; Burns, J.C. Global gene expression profiling identifies new therapeutic targets in acute Kawasaki disease. *Genome Med.* **2014**, doi:10.1186/s13073-014-0102-6.
3. Herberg, J.A.; Kaforou, M.; Gormley, S.; Sumner, E.R.; Patel, S.; Jones, K.D.J.; Paulus, S.; Fink, C.; Martinon-Torres, F.; Montana, G.; et al. Transcriptomic profiling in childhood H1N1/09 influenza reveals reduced expression of protein synthesis genes. *J. Infect. Dis.* **2013**, *208*, 1664–1668, doi:10.1093/infdis/jit348.
4. McCrindle, B.W.; Rowley, A.H.; Newburger, J.W.; Burns, J.C.; Bolger, A.F.; Gewitz, M.; Baker, A.L.; Jackson, M.A.; Takahashi, M.; Shah, P.B.; et al. Diagnosis, treatment, and long-term management of Kawasaki disease: A scientific statement for health professionals from the American Heart Association. *Circulation* **2017**, *135*, e927–e999, doi:10.1161/CIR.0000000000000484.
5. Cox, J.; Mann, M. MaxQuant enables high peptide identification rates, individualized p.p.b.-range mass accuracies and proteome-wide protein quantification. *Nat. Biotechnol.* **2008**, *26*, 1367–1372, doi:10.1038/nbt.1511.
6. Cox, J.; Hein, M.Y.; Luber, C.A.; Paron, I.; Nagaraj, N.; Mann, M. Accurate proteome-wide label-free quantification by delayed normalization and maximal peptide ratio extraction, termed MaxLFQ. *Mol. Cell. Proteomics* **2014**, *13*, 2513–2526, doi:10.1074/mcp.M113.031591.
7. Supek, F.; Bošnjak, M.; Škunca, N.; Šmuc, T. REVIGO Summarizes and Visualizes Long Lists of Gene Ontology Terms. *PLoS One* **2011**, *6*, e21800, doi:10.1371/journal.pone.0021800.
8. Tibshirani, R. Regression Shrinkage and Selection via the Lasso. *J. R. Stat. Soc. Ser. B* 1996, *58*, 267–288.
